# Therapeutic potential of DNA methyltransferase inhibitors with immune checkpoint inhibitor therapy in breast cancer

**DOI:** 10.15698/cst2018.03.129

**Published:** 2018-03-07

**Authors:** Na Luo, Ayaka Sugiura, Justin M. Balko

**Affiliations:** 1 Department of Anatomy and Histology, School of Medicine, Nankai University, Tianjin, China.; 2 Department of Pathology, Microbiology and Immunology, Vanderbilt University Medical Center, Nashville TN 37232, USA.; 3 Department of Medicine, Vanderbilt University Medical Center, Nashville TN 37232, USA.; 4 Cancer Biology Program, Vanderbilt University, Nashville TN 37232, USA.; 5 Breast Cancer Research Program, Vanderbilt University, Nashville TN 37232, USA.

**Keywords:** epigenetic, DNA methyltransferase, immunotherapy, PD-L1, NFkB, cytotoxic T cells, MHC

## Abstract

However, ICI therapy has thus far demonstrated limited efficacy in breast cancers, where tumor mutation rates are intermediate. Nonetheless, because of limited but positive signals in early trials, combinations of therapies to enhance anti-tumor immunity, and thus response to ICIs in breast cancer, are actively being sought. Our laboratory recently found that guadecitabine, a next-generation DNA methyltransferase inhibitor (DMTi), potentiated cytotoxic CD8^+^ T cell responses in breast cancer, which appeared to occur by the following mechanisms: (1) DMTi treatment hypomethylated and up-regulated both baseline and IFN-γ-induced MHC-I expression, thereby enhancing antigen presentation capacity, (2) DMTi treatment increased Cxcr3 ligands/chemokines (i.e., *Cxcl9*, *Cxcl10*, and *Cxcl11*) expression and recruited cytotoxic CD8^+^ T cells into the tumors and (3) DMTi treatment activated NFκB signaling, presumably through the expression of endogenous retroviral (ERV) sequences in tumor cells, initiating an innate response observed in other solid tumor types [Luo et al., Nat Commun 9(1):248]. Most importantly, DMTi treatment primed breast cancer and improved responses to anti-PD-L1 therapy.

Tumor infiltrating lymphocytes (TILs) are a positive prognostic biomarker in breast cancer, particularly in hormone receptor-negative disease. High TILs associate with an improved survival rate in breast cancer patients, and it is these patients who also appear to be most likely to benefit from immunotherapies targeting the PD-1/L1 axis, based on early correlative data. Presumably, these associations are indicative of ongoing anti-tumor immunity which relies on the PD-1/L1 axis for tumor maintenance. However, given the limited single-agent activity of anti-PD-1/L1 therapy in metastatic breast cancer, many translational studies are aimed at enhancing this response rate through concomitant therapies to stimulate adaptive immunity in the tumor.

Anti-tumor T-cell mediated immunity can be modulated by a number of factors, such as abundance of non-self neoantigens, antigen processing and presentation, T cell recognition, and the activation/exhaustion or fitness state of the T cell population. Furthermore, signals of inflammation, such as chemokines must be released from the tumor microenvironment to promote T cell trafficking from lymphoid organs. Each of these factors are potential targets for enhancing anti-tumor immunity, and their contributions are likely to be tumor- or patient-specific.

One recently proposed strategy for enhancing tumor inflammation is through the use of epigenetic modulation. Epigenetic alterations play an important but poorly-understood role in the initiation and progression of many cancer types, including those of the breast. For instance, epigenetic marks (e.g. promoter CpG methylation) that are tightly controlled during cell fate decisions can become deregulated in cancer, resulting in silencing of tumor suppressor functions or re-expression of genes normally confined to other cell types and lineages. As such, hypermethylation mediated by DNA methyltransferases (DNMTs) can silence the expression of certain tumor suppressor genes. Conversely, the use of DNMT inhibitors (DMTi) to derepress these marks has been widely studied, and is an efficacious therapy in some tumor types, primarily myeloid diseases. However, many additional mechanisms of action of these agents are now being appreciated, including their ability to promote tumor inflammation through expression of ERV sequences that are normally latent in ~5-8% of the genome.

In our recent work, we found an inverse correlation between methylation levels of MHC-I and MHC-II gene promoter regions and MHC-I and MHC-II mRNA expression in human breast cancers. Methylation levels of MHC-I and MHC-II were also inversely correlated with *CD8A* (predominantly a cytotoxic T cell surrogate marker) mRNA expression, suggesting an association of MHC expression and methylation with immunogenicity. Using murine mammary tumor cells, we confirmed that methylation of the MHC-I gene can be a rate-limiting step in its expression, and DMTi treatment can de-methylate the MHC-I promoter region to increase its expression in cancer. MHC-I presents processed antigens to CD8^+^ T cells and its components [HLA-A, -B, -C and beta-2-microglobulin (B2M)] are often positively associated with T cell activity. The work of others have shown that somatic mutations in MHC-I genes result in defects in antigen presentation and promote resistance to immune checkpoint inhibitor therapy. For example, loss of heterozygosity in *B2M* is enriched in patients who fail to respond to ICI, while homozygous deletion of *B2M* was identified in patients with acquired resistance to ICIs or personalized RNA vaccines. *In vivo*, DMTi treatment also enhanced MHC-I expression on tumor cells, while suppressing tumor growth and inducing an immunologic response as evidenced by enhanced CD8+ T cell infiltrate both on immunohistochemistry analysis and CD8+ immuno-PET (positron-emission tomography).

In cell line studies, we also observed that DMTi treatment up-regulated MHC-I expression in response to IFN-γ stimulation, a potent T-cell secreted inflammatory cytokine that is becoming increasingly implicated in the anti-tumor immune process and response to ICI therapy. DMTi treatment also significantly increased IFN-γ-induced Cxcr3 chemokine (*Cxcl9/10/11*) expression. Cxcr3 is highly expressed by Th1 cells, cytotoxic CD8^+^ T cells, natural killer (NK) cells, and NK-T cells. Upon drainage of inflammatory Cxcr3 ligands to the lymph node, Cxcr3 ligation leads to T cell trafficking to the tumor (or other inflammation sites). This promotes Th1 polarization and contributes to heightened cytotoxic activity by CD8+ T cells, which is required for the anti-tumor response pursuant to ICI therapy.

To better understand how DMTi treatment enhanced IFN-γ-mediated MHC-I and Cxcr3 ligand expression, we explored cell signaling pathways modulated by DMTi treatment in breast cancer cells. DMTi treatment activated NFκB activity, but this effect was required only for IFN-γ-induced MHC-I and Cxcr3 ligand expression, not direct MHC-I upregulation. Accumulating evidence shows that epigenetic agents, including both histone deacetylase inhibitors and DMTis, can up-regulate the expression of ERV double-stranded RNA (dsRNA). ERV dsRNA is sensed by pathogen recognition receptors in the cytoplasm and can activate the NFκB transcription factor. This results in the expression of Type I interferons which play a central role in the regulation of innate immune response and antitumor immunity. Thus, DMTi treatment may have multiple effects on tumor-immune interplay - both direct DNA demethylation of MHC-I, as well as activation of innate immune responses through ERV dsRNA expression and Type I inflammatory responses.

Given the observed effects of DMTi treatment on tumor cell inflammation and antigen presentation, our work culminated in the evaluation of whether this induction of anti-tumor immunity could potentiate the response to anti-PD-L1 ICI therapy. To test this, we utilized two murine breast cancer models (MMTV-neu and polyoma V middle T; PyVmT). In both models (widely consider luminal-like models of breast cancer), we found that DMTi treatment could enhance response to anti-PD-L1 immunotherapy. It also has been reported that DNA-demethylating agents primes or sensitizes pre-clinical melanoma, lung cancer, and colorectal cancer models to ICI therapy. Therefore, our work provides the preclinical rationale to suggest that a clinical trial in luminal-like breast cancer combining next-generation DMTis, such as guadecitabine, with anti-PD-1/L1 immunotherapy should be conducted.

In summary, we found that DMTi treatment in breast cancer amplifies cytotoxic CD8^+^ T cell responses with at least 3 possible contributing mechanisms of action (**Fig 1**). These findings *in toto* permit speculation on the possibility that DMTi treatment primes breast cancer cells to elicit a positive response to ICI therapy. Thus, novel clinical trials combining these agents in breast cancer therapy are warranted to determine if patient benefit can be extrapolated from our results, and those of others in the field. Finally, evaluation of combinations of epigenetic modulators (*e.g.* those inhibiting EZH2, which have been shown to enhance MHC-II expression) may produce more profound immunologic effects, and studies evaluating these combinations are underway.

**Figure 1:**
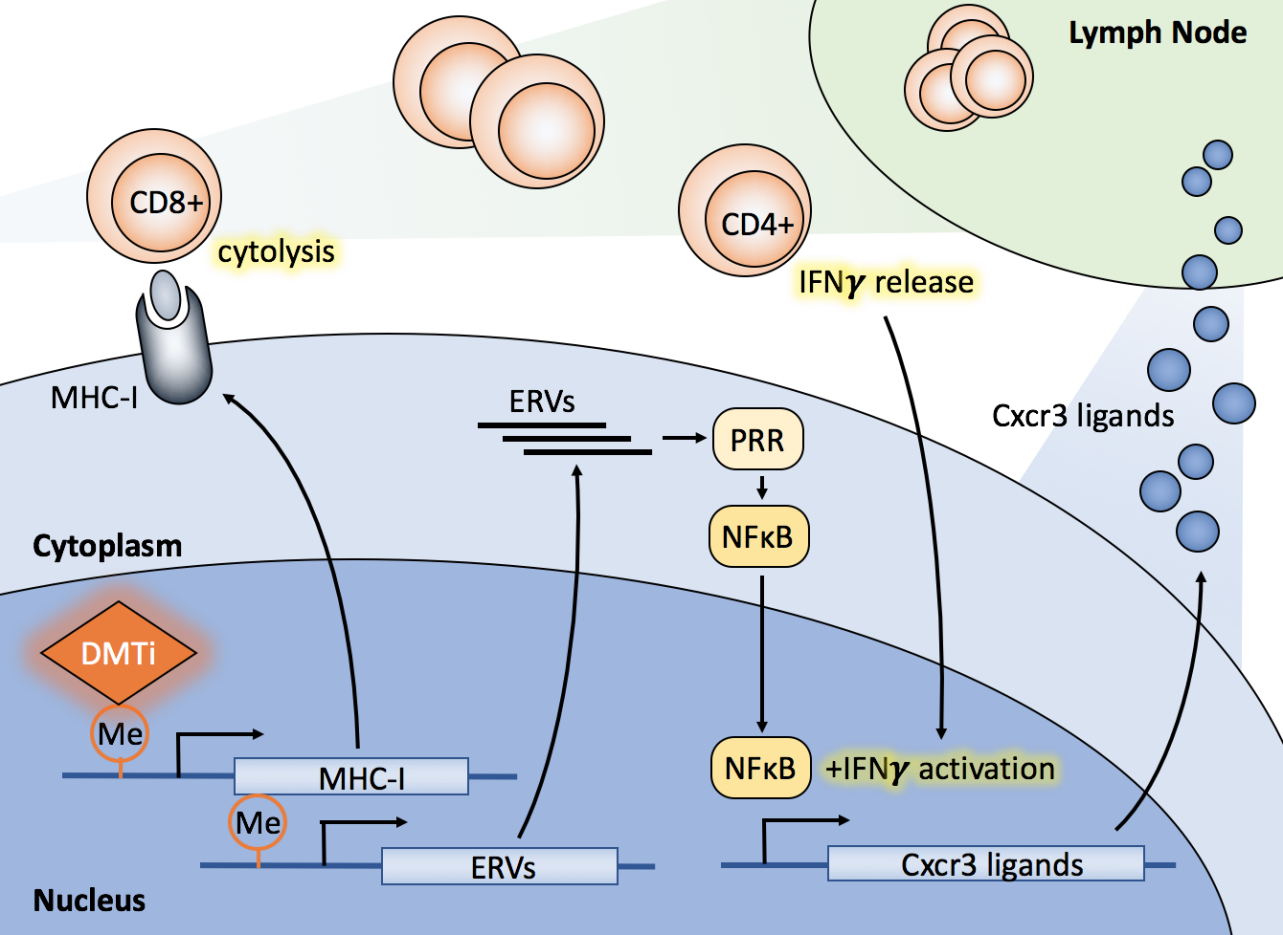
FIGURE 1: Multiple mechanisms of DMTi potentiation of anti-tumor immunity. First, DMTi treatment up-regulates baseline MHC-I expression and MHC-I expression in response to IFN-γ, thereby enhancing antigen presentation capacity and cytotoxic CD8^+^ T cell activation. Second, DMTi treatment increases Cxcr3 chemokine (i.e., *Cxcl9, Cxcl10, Cxcl11*) expression, thereby enhancing T cell trafficking to the tumor microenvironment. Third, DMTi treatment activates NFκB signaling, presumably through ERV dsRNA expression, initiating an innate immune response. Each of these mechanisms *in toto* likely contribute to enhanced response to ICI therapy.

